# Marine Compounds and Age-Related Diseases: The Path from Pre-Clinical Research to Approved Drugs for the Treatment of Cardiovascular Diseases and Diabetes

**DOI:** 10.3390/md22050210

**Published:** 2024-05-03

**Authors:** Maria Elisa Giuliani, Giorgia Bigossi, Giovanni Lai, Serena Marcozzi, Dario Brunetti, Marco Malavolta

**Affiliations:** 1 Advanced Technology Center for Aging Research and Geriatric Mouse Clinic, IRCCS INRCA, 60121 Ancona, Italy; m.giuliani@inrca.it (M.E.G.); g.bigossi@inrca.it (G.B.); g.lai@inrca.it (G.L.); s.marcozzi@inrca.it (S.M.); 2 Unit of Medical Genetics and Neurogenetics, Fondazione IRCCS Istituto Neurologico “Carlo Besta”, 20126 Milano, Italy; dario.brunetti@istituto-besta.it; 3 Department of Clinical Sciences and Community Health, University of Milan, 20122 Milan, Italy

**Keywords:** ageing, cardiovascular disease, type 2 diabetes mellitus, marine compounds, in vivo studies, clinical trials

## Abstract

Ageing represents a main risk factor for several pathologies. Among them, cardiovascular diseases (CVD) and type 2 diabetes mellitus (T2DM) are predominant in the elderly population and often require prolonged use of multiple drugs due to their chronic nature and the high proportion of co-morbidities. Hence, research is constantly looking for novel, effective molecules to treat CVD and T2DM with minimal side effects. Marine active compounds, holding a great diversity of chemical structures and biological properties, represent interesting therapeutic candidates to treat these age-related diseases. This review summarizes the current state of research on marine compounds for the treatment of CVD and T2DM, from pre-clinical studies to clinical investigations and approved drugs, highlighting the potential of marine compounds in the development of new therapies, together with the limitations in translating pre-clinical results into human application.

## 1. Introduction

The rapid global rise in the elderly population and in life expectancy [1,2] underscores the necessity to find new strategies to improve the health of older individuals. This includes a continuous pursuit of new molecules to effectively target the main age-related pathologies with minimal side effects.

In this context, interesting drug candidates could come from the sea, representing a large reservoir of structurally different molecules with unique chemical features derived from the exceptionally high biodiversity of the marine environment. This allows for a high diversity of mechanisms of action and, consequently, of biological targets. Among the plethora of marine molecules, different mechanisms of action and pharmacological properties have been described, which are periodically updated and range from antibacterial, antifungal, antiprotozoal, antituberculosis, antiviral, antidiabetic, anti-inflammatory, and anticancer activities to those affecting the immune and nervous systems, as well as numerous miscellaneous mechanisms of action [3].

Since the first marine molecule was commercialised in 1969 for the treatment of leukaemia (i.e., cytarabine, derived from a marine sponge), a total of thirteen sea-derived drugs have been approved in the EU and/or USA, most of which target tumour pathologies (ten), and the remaining ones are used to treat viral infections, chronic pain, and hypertriglyceridemia [4]. Moreover, several marine-derived compounds are currently under clinical trials, namely four in Phase III, eight in Phase II, and twenty in Phase I [4].

This review summarises the current advancement in the field of marine bioactive compounds for the treatment of two of the main age-related diseases, cardiovascular diseases (CVD) and Type 2 diabetes mellitus (T2DM). These pathologies are closely related since they display common risk factors such as obesity, dyslipidemia, inflammation, and ageing. Moreover, T2DM could predispose to the development of CVD, and patients affected by T2DM develop cardiovascular problems in a higher proportion than normoglycemic people [5]. The review will consider the potentiality of marine molecules at various stages of the research and approval process, from in vivo pre-clinical studies to more advanced stages, including molecules entered into clinical trials and approved drugs. Finally, common mechanisms of action will be discussed, as well as the challenges to face in accelerating the translation process towards clinical application.

## 2. Marine Compounds and Cardiovascular Disease

Ageing is characterised by a decline in cardiovascular functionality, including altered function of the left ventricle, decreased heart rate, arrhythmias, cardiac hypertrophy and fibrosis, increased stiffness and thickening of arterial vessels, and endothelial dysfunction [6]. The frequency of CVD rises with advanced age, with an incidence of 35–40% in 40–60-year-old people, 77–80% in 60–80-year-old people, and over 85% in people >80 years old [7]. CVD is still the main cause of death in people over 70 worldwide [8]. Ageing is the primary cause of age-related CVD, serving as an independent risk factor for the development of atherosclerosis, thrombosis, myocardial infarction, stroke, and coronary artery disease [9,10,11]. Hypertension and hyperlipidemia, which are other important risk factors for CVD, are also strongly associated with ageing [12,13]. Currently, the most common and effective drugs for the treatment of CVDs are statins (used to lower cholesterol and prevent atherosclerosis), the anticoagulant heparin, and anti-hypertensives such as angiotensin-I-converting enzyme (ACE) inhibitors, calcium channel blockers, and β-adrenergic blocking agents. However, their use is not devoid of adverse effects [14]. Hence, the discovery of alternative therapies with reduced side effects is desirable, leading to an increasing interest in the efficacy of marine-derived compounds against CVDs. In vitro studies demonstrated hypolipidemic, anti-hypertensive, anticoagulant, and antiplatelet effects for many marine molecules, including organic small molecules, lipids, bioactive peptides, and sulfated polysaccharides [15,16,17]. Marine products with anti-atherosclerotic, anti-thrombotic, and cardiovascular protective effects in vivo, as well as anti-hypertensive and hypolipidemic activities, will be discussed in this paragraph.

### 2.1. Pre-Clinical In Vivo Studies on Marine Compounds for CVDs Treatment

Increasing pre-clinical studies have demonstrated the efficacy of a considerable number of marine compounds in the treatment of atherosclerosis, being able to significantly reduce atherosclerotic plaque size and/or progression in mouse, rat, or rabbit models [18,19] (Table 1). Such an anti-atherosclerotic effect was often associated with a reduction in plasmatic levels of lipids and/or pro-inflammatory molecules, but the upstream mechanism of action is likely different for each of these compounds. Among them, the most widely studied are undoubtedly the polysaccharides fucoidan, derived from brown algae, and the xanthophyll carotenoid astaxanthin, mostly derived from microalgae, krill, and crustaceans. The fucoidan effect against atherosclerosis was extensively proved in mice by distinct studies, where attenuation of atherosclerosis was paralleled by reduced serum lipid levels, inflammation, and oxidative stress [20,21,22,23]. The proposed mechanism of action of fucoidans includes anticoagulant, antithrombotic, antioxidant, anti-inflammatory, cholesterol-lowering, and endothelial protection activities [24]. However, when orally administered, most of these effects can be mediated by their capacity to modulate the intestinal microbiota composition and nutrient absorption [25]. Fucoidan can also induce autophagy in foam cells, which are implicated in the development of atherosclerotic plaques, consequently decreasing their buildup of pro-atherogenic lipids [26]. The anti-atherosclerotic effect of the carotenoid astaxanthin was demonstrated in different animal models, showing a reduction in the area of aortic atherosclerotic plaques in rats and mice [27,28], as well as plaque stabilisation in rabbits [29], with improvements in lipid metabolism, inflammation, and oxidative stress. Astaxanthin can integrate into cell membranes, offering stability and direct protection against oxidative damage [30]. Astaxanthin not only exerts its influence on multiple biological defence mechanisms through its potent antioxidant activity but also plays a role in maintaining and augmenting mitochondrial function. This is achieved by directly modulating the AMPK/sirtuins/PGC-1α pathway, among others [31,32].

More recently, the sea cucumber saponins have received considerable attention as anti-atherogenic marine compounds, promoting plaque regression and exerting a lipid-lowering effect in mice [33,34]. The amphipathic nature of saponins enables them to directly interfere with the uptake and processing of lipids and to interact with cell membranes, disrupting their structure and altering their permeability [35]. Likely, sea cucumber saponins may be able to target cells involved in inflammation and the formation of atherosclerotic plaque; indeed, some saponins were observed to reduce cholesterol esters content in macrophage foam cells [36].

Marine-derived molecules capable of reducing atherosclerotic lesions in vivo include a diverse array of chemical structures and mechanisms of action. Manzamine A, an alkaloid sourced from marine sponges, showed acyl-coenzyme A:cholesterol acyl-transferase (ACAT) inhibitory activity and reduced cholesterol ester accumulation in macrophages, attenuating the formation of foam cells [37]. Further, it displays root mechanisms such as antiproliferative and cytotoxic effects [38]. Manzamine A has been shown to decrease the level of Bcl-2, causing mitochondrial membrane potential (Δψm) loss and enhancing the activity of caspase-3 and caspase-7, inducing the release of CytC in HCT116 cells [39,40]. Note that manzamine A also presents anti-viral effects [36], which is particularly interesting considering that age-related cardiovascular diseases are frequently reported to be associated with certain bacterial and viral infections [41]. Conversely, saringosterol, a phytosterol derived from the marine algae *Sargassum fusiforme*, acts as a potent activator of the liver X receptor β, involved in cholesterol absorption, transport, and elimination [42]. Thus, it may impact the metabolism of lipids with a distinctive cellular target and specific metabolic transformation, inducing further pharmacological effects.

Asperlin, mycoepoxyedien, and xyloketal B, representing polyketide secondary metabolites and a polyphenolic compound, are all derived from marine fungi. The first two compounds, known for their antiviral and cytotoxic activities, exert an anti-inflammatory effect that inhibits the formation of macrophage foam cells [43,44]. Xyloketal B, known for its antioxidant and anti-inflammatory properties, showed endothelial-protecting activity through the regulation of the Akt/eNOS pathway [45]. In an in vitro model using human umbilical vein endothelial cells (HUVECs) to mimic oxidised low-density lipoprotein (oxLDL)-induced endothelial injury, pre-treatment with xyloketal B greatly reduced the formation of superoxide anion generated by oxLDL as well as the mRNA expression of the NADPH oxidase subunits gp91phox and p47phox [36,46]. These results suggest that xyloketal B reduces the generation of reactive oxygen species (ROS) by blocking the action of NADPH oxidase and reducing the expression of its subunits on mRNA. Similarly, xyloketal B stimulated the release of NO by re-establishing the equilibrium between ROS and NO, which in turn prevented the formation of peroxynitrite after oxLDL damage.

Caulerpin, a secondary metabolite isolated from the invasive algae *Caulerpa cylindracea*, has been suggested as a possible hypolipidemic and anti-atherogenic compound. An in silico analysis demonstrated that caulerpin is an agonist of the peroxisome proliferator activated receptor alpha (PPARα), which is a key regulator of lipid metabolism, and caulerpin was able to upregulate PPARα target genes in vitro and in vivo [47].

Also, peptides from salmon protein hydrolysate (SPH) contribute to this varied arsenal of bioactive anti-atherosclerotic compounds [48]. Parolini and coworkers demonstrated that SPH supplementation is able to reduce atherosclerotic plaque area in apo E^−/−^ mice fed with a high fat diet, reducing the plasma concentrations of IL-1β, IL-6, TNF-α, and GM-CSF, whereas plasmatic triacylglycerols and cholesterol remained unaltered, as well as mitochondrial fatty acid oxidation or ACAT activity. This study demonstrated that the SPH diet, which acts at both the vascular and systemic levels, decreases atherosclerosis without a direct correlation with changes in plasma lipids or fatty acids, but is able to influence the inflammatory responses [48].

The marine environment offers a plethora of molecules that are interesting for the development of novel antithrombotic drugs (Table 1). Antithrombotics include anticoagulants (that prevent fibrin strand formation), anti-platelets (that inhibit platelet aggregation), and fibrinolytic/thrombolytic (that dissolve the thrombus once formed) drugs; their major side effect still remains the augmented risk of bleeding. Intravenous administration of fucoidan as well as oral administration of its low-weight modified form were shown to have anticoagulative and antithrombotic effects, preventing microvascular thrombus formation and delaying complete vascular occlusion in mice, with no obvious side effects as well [23,49]. This is not surprising, since fucoidan shows a high affinity for fibroblast growth factor-2 (FGF-2, a potent atherogenic factor) like heparin and thus can protect FGF-2 from inactivation [50]. Some sulfated glycans from algae or sea urchin species (in particular 2-sulfated galactan) demonstrated an antithrombotic effect in rats by promoting the reduction of thrombus weight, together with anticoagulant and anti-platelet properties in vitro, without side effects such as hypotension and bleeding [51]. Several studies suggest that their main mechanism of action may involve the direct inhibition of coagulation factors [51,52].

Inhibition of thrombosis and antiplatelet properties were also demonstrated in mice for the alkaloid fascaplysin (derived from a marine sponge), the triterpenoid frondoside A (a saponin from sea cucumbers), and the crab peptide tachyplesin I, the latter with no observed toxicity [53,54,55]. Fascaplysin and frondoside A reduce platelet activation by inhibiting the PI3K/Akt pathway and glycoprotein (GP)IIb/IIIa [53,54], and frondoside A is also known to stimulate the lysosomal activity of macrophages [56]. Tachyplesin I, in addition to inhibiting platelet aggregation and thrombosis by interfering with the PI3K/AKT pathway, is also widely studied for its antiviral and antimicrobial effects, which may be relevant considering that thrombosis is also a common consequence of infections [55,57].

The marine yeast-derived *R-/S-*2-(2-Hydroxypropanamido) benzoic acid (*R-/S-*HPABA) and the extracts of the alga *Eisenia bicyclis* were shown to reduce thrombus weight in rats and/or mice and exert a strong inhibition of platelet aggregation in vitro [58,59]. These effects appear to be mediated through distinct mechanisms: *R-/S-*HPABA likely inhibits cyclooxygenase-1 (COX-1) activity and thromboxane B2 (TXB2) formation, while *Eisenia* extracts reduce P2Y_12_ downstream signalling, a process crucial in platelet activation and aggregation [58,59].

Recently, a novel marine protease called SK was isolated from the marine worm *Sipunculus nudus*. SK, which belongs to the serine protease family, reduced thrombus weight in rats by exerting fibrinolytic and fibrinogenolytic activities and inhibiting platelet aggregation [60].

Marine compounds may also have a role in the treatment of myocardial infarction (Table 1). Omega-3 polyunsaturated fatty acids (PUFA) from fish oil (eicosapentaenoic, EPA, and/or docosahexaenoic acid, DHA) were shown to reduce the infarct size in rats, rabbits, and pig models [61], while a pre-treatment with omega-3 from krill oil reduced left ventricle dilatation and remodelling after induction of myocardial infarction in rats [62]. These long-chain polyunsaturated fatty acids can stabilise the cell membrane and neutralise extracellular ROS through their conjugated double bonds [63]. These properties also underlie their potential for providing endothelial protection and manifesting anti-inflammatory properties.

Echinochrome A, a pigment found in sea urchin needles (registered in the Russian pharmacopoeia as Histochrome^®^), prevented chronic heart failure (left ventricle dysfunction) after myocardial infarction in mice [64] and attenuated myocardial ischemia/reperfusion injury and cerebral ischemic injury in rats [65,66]. This effect has been partly attributed to the prevention of sulphide catabolism-mediated oxidative stress [64] and to the reduction of fibrosis area [67]. Further, it was reported that echinochrome A effectively boosted mitochondrial mass and oxidative phosphorylation in rat cardiomyoblast H9c2 cells, leading to a notable enhancement in mitochondrial energy efficiency. This improvement was attributed to the modulation of key regulatory genes involved in mitochondrial biogenesis, such as PGC-1α and NRF-1 [68]. Moreover, echinochrome A possesses therapeutic potential to mitigate the adverse cardiotoxic effects induced by clinically utilised drugs such as SNP and Dox. It was reported that echinochrome A effectively prevented mitochondrial dysfunction and the activation of MAPK cell death signalling pathways triggered by the administration of cardio/mitotoxic drugs in rat cardiac myoblast H9c2 cells and isolated rat cardiomyocytes [69].

As regards hypertension, many marine peptides have shown ACE-inhibitory activity in vivo without evident side effects [70] (Table 1). Peptides from hydrolysates of tuna muscle or frame, sea bream scales, *Styela clava* tunicate, *Acaudina molpadioidea* sea cucumber, and from the algae *Gracilariopsis lemaneiformis* and *Undaria pinnatifida* were all able to decrease blood pressure in a spontaneously hypertensive rat model [71,72,73,74,75,76,77].

Hypertensive rat treatment with fucoidan resulted in a persistent reduction of high blood pressure, with mechanisms that might involve an endothelial-protective function mediated by the Akt-eNOS signalling pathway [78]. In addition, the algal polysaccharides alginate, in both potassium and sodium form, has established anti-hypertensive activity, as demonstrated in spontaneous and induced rat models of hypertension [79,80,81,82,83], both as a scaffold for potassium delivery [79] and as an active molecule [80,81,82,83]. This effect was associated with low cardiovascular and renal damage [81] and downregulation of heart failure markers [82], and is likely due to a modulation of the gut microbiota and an improvement of the gut barrier [82,83] (Table 1).

**Table 1:** Marine compounds showing cardiovascular effects in pre-clinical in vivo studies and related mechanisms of action.

Marine Compound	Source	CVD Model	Effects	Mechanisms	Positive Control	Ref.
Astaxanthin	Microalgae, crustaceans	Rat, high fat diet; Mice, ldlr^−/−^ and Apoe^−/−^; Rabbits, Watanabe heritable hyperlipidemic	Anti-atherosclerotic, hypolipidemic, atherosclerotic plaque stabilization	Decrease macrophage infiltration decrease apoptosis, antioxidant	Atorvastatin	[27,29]
Manzamine A	Sea sponge *Acanthostrongylophora ingens*	Mice, Apoe^−/−^	Anti-atherosclerotic, hypolipidemic	Inhibition of ACAT cholesterol esters decrease (macrophages), foam cell formation decrease	Absent	[37]
Saponins	Sea cucumber	Mice, Apoe^−/−^	Anti-atherosclerotic, hypolipidemic	Regulation of hepatic cholesterol efflux, change in microbiota, anti-inflammatory	Simvastatin	[33,34]
Saringosterol	Brown algae *Sargassum fusiforme*	Mice, Apoe^−/−^	Anti-atherosclerotic, hypolipidemic	LXRβ inhibition, cholesterol efflux increase, foam cell formation decrease, choloesterol catabolism increase	T0901317 (LXR agonist)	[42]
Xyloketal B	Marine fungus *Xylaria* sp.	Mice, Apoe^−/−^	Anti-atherosclerotic, endothelial function improvement	Regulation of the Akt/eNOS pathway, decrease vascular oxidative stress	Simvastatin	[45]
Mycoepoxydien	Marine fungus *Diaporhte* sp.	Mice, Apoe^−/−^	Anti-atherosclerotic	Foam cell formation decrease, NF-κB pathway inhibition, anti-inflammatory	Absent	[44]
Asperlin	Marine fungus *Aspergillus versicolor*	Mice, Apoe^−/−^	Anti-atherosclerotic	Cholesterol efflux increase (macrophages), foam cell formation decrease, anti-inflammatory	Simvastatin	[43]
Fascaplysin	Sponge *Fascaplysinopsis*	Mice, photochemically-induced thrombosis	Antithrombotic, antiplatelet	Inhibition of PI3K signalling and glycoprotein IIb/IIIa	Heparin	[54]
Frondoside A	Sea cucumber *Cucumaria frondosa*	Mice, photochemically-induced thrombosis	Antithrombotic	Inhibition of PI3K/Akt signalling and glycoprotein IIb/IIIa	Clopidogrel	[53]
Tachyplesin I	Crab *Tachypleus tridentatus*		Antithrombotic, antiplatelet	Regulation of PI3K/Akt signalling	n.a.	[55]
*R-/S-*2-(2-Hydroxypropanamido) benzoic acid (*R-/S-*HPABA)	Marine fungus *Penicillium chrysogenum*	Mice, collagen-epinephrine induced thrombosis; Rats, carotid artery-induced thrombosis	Antithrombotic, antiplatelet	COX1 inhibition, TXB2 decrease	Aspirin	[58]
Echinochrome A	Sea urchins	Rat, middle cerebral artery occlusion model; Rats, myocardial ischemia-reperfusion model; Mice, coronary artery ligation	Cerebral infarct volume reduction, cardioprotective (reduced infarct size, heart fibrosis, remodeling and dysfunction)	Regulation of Akt/ERK pathway and BDNF, regulation of apoptosis and ferroptosis, antioxidant, prevent reactive sulfur species catabolism	Absent	[64,65,66]
Fucoidan	Brown algae e.g., *Saccharina japonica, Undaria pinnatifida*	Hyperlipidemic mice, P407-induced; Mice, Apoe*^shl^* (spontaneously hyperlipidemic); Mice, ldlr^−/−^; Mice, photochemically-induced thrombosis; Rats, l-NAME-induced hypertensive; Mice, Apoe^−/−^	Anti-atherosclerotic, hypolipidemic, hepatic steatosis reduction, endothelium-protective, anti-thrombotic, anticoagulative, anti-hypertensive	Regulation of hepatic SREBP-2, PPARa pathway activation, antioxidant (inhibition of eNOS, NOX-4, ICAM-1, VCAM-1), PI3K/Akt/eNOS pathway activation, FGF and VEGF pathways regulation, inhibition of vascular cells proliferation, anti-inflammatory	Atorvastatin, probucol, heparin	[20,21,22,23,49,50,78]
Sulphated glycans	Sea urchin *Lytechinus variegatus, Echinometra lucunter*	Rats, tromboplastin-induced thrombosis	Anti-thrombotic, anticoagulative	Unidentified	Heparin	[51]
Potassium alginate	Brown algae	Rats, DOCA salt-induced hypertensive; Rats, spontaneously hypertensive	anti-hypertensive	Increased potassium levels and sodium excretion, decreased angiotensin II and natriuretic peptide levels, modulation of gut microbiota	KCl, captopril	[79,82]
Sodium alginate	Brown algae *Saccharina japonica*	Rats, spontaneously hypertensive; Rats, salt-induced; Rats, renovascular hypertensive (2K1C)	Anti-hypertensive, prevent kidney damage, reduced cardiac fibrosis	Decrease fractional sodium excretion, modulation of gut barrier	Absent	[80,81,83]
Protease	Marine worm *Sipunculus nudus*	Rats, FeCl_3_-induced thrombosis	Antithrombosis, anticoagulant	Fibrinolytic and fibrinogenolytic activities	Urokinase	[60]
Salmon protein hydrolysate	Fish (salmon)	Mice, Apoe^−/−^	Anti-atherosclerotic	Anti-inflammatory	Absent	[48]
Peptides	Brown algae *Undaria pinnatifida*, Sea bream scale, Tuna dark muscle, Tunicate *Styela clava*, Sea cucumber *Acaudina molpadioidea*, Tuna frame, Red algae *Gracilariopsis lemaneiformis*	Rats, spontaneously hypertensive	Anti-hypertensive	ACE inhibition	Captopril, enalapril	[71,72,73,74,75,76,77]
EPA	Fish	Rabbits, myocardial ischemia-reperfusion model	Cardioprotective (reduced infarct size)	Opening Ca-activated K channels	Absent	[84]
DHA	Fish	Pigs, myocardial ischemia-reperfusion model	Cardioprotective (reduced infarct size, reduced mortality)	n.a.	Absent	[85]
Omega-3 PUFA	Fish	Rats, myocardial ischemia-reperfusion model	Cardioprotective (reduced infarct size)	Activation of Akt pathway, reduced apoptosis		[86]
EPA or DHA	Fish	Rats, myocardial ischemia-reperfusion model	Cardioprotective (reduced infarct size)	Activation of Akt pathway, reduced caspase-3-activity, inhibition of mPTP channel opening		[87]
Omega-3 PUFA	Krill	Rats, myocardial ischemia induction	Cardioprotective (reduced left ventricle remodeling and hyperthrophy)	Anti-inflammatory	Absent	[62]
Extract	Brown algae *Eisenia bicyclis*	Rats, arteriovenous (AV)-shunt model	Antithrombotic, antiplatelet	P_2_Y_12_ signaling inhibition, PI3K/Akt signalling inhibition, integrin αIIbβ3 signalling inhibition	Absent	[59]

ACAT—acyl-coenzyme A:cholesterol acyl-transferase; ACE—angiotensin converting enzyme; COX1—cyclooxigenase 1; DOCA—deoxycorticosterone acetate; eNOS—endothelial nitric oxide synthase; NOX-4—NADPH oxydase 4; ICAM-1—intercellular adhesion molecule-1; LXR-β—liver X receptor beta; mPTP—mitochondrial permeability transition pore; PI3K—phosphatidyl inositol 3-kinase; NF-κB—nuclear factor kappa B; FGF—fibroblast growth factor; VEGF—vascular endothelial growth factor; BDNF—brain-derived neurotrophic factor; SREBP-2—sterol regulatory element-binding protein 2; PPARα—peroxisome proliferator activated receptor alpha; ERK—extracellular signal-regulated kinase; TXB2—thromboxane B2; VCAM-1—vascular cell adhesion molecule-1.

### 2.2. Marine Compounds under Clinical Trial for CVDs Treatment

Only a few molecules among those under pre-clinical studies have undergone clinical investigations for CVD treatment so far [17]. The most explored molecules are fish-derived omega-3 PUFA, i.e., EPA and DHA. Several clinical trials were conducted or are ongoing to evaluate their benefits on cardiovascular health, highlighting contrasting results: while some studies highlighted a lower risk of adverse cardiovascular events after omega-3 PUFA treatment, other studies showed no beneficial effects on cardiovascular health [88]. It was suggested that the effects of EPA alone are more beneficial than those of combined formulations (EPA + DHA) [89,90].

Alginate was investigated for cardiac regeneration after a heart injury [89,90]. The IK-5001 alginate hydrogel formulation was tested in clinical trials to evaluate the effects of the intracoronary injection on the prevention of negative ventricular remodeling. Despite the good tolerability of the alginate implant [91], a multicenter clinical trial involving individuals with recent myocardial infarction revealed no beneficial effects on left ventricle remodelling or cardiac events (Clinical Trial Identifier: NCT01226563) [92]. On the contrary, promising results were obtained for the Algisyl-LVR™ alginate hydrogel implants after intramyocardial injection. The results of the multicenter randomised clinical trial demonstrated that Algisyl therapy improved functional capacity and clinical outcomes in patients with advanced heart failure compared to standard medical therapy (NCT01311791) [93].

The carotenoid astaxanthin is also under clinical investigation as a food supplement, formulated in combination with other molecules or administered alone, for the treatment of arrhythmias or strokes (ongoing clinical trials NCT02087033, NCT01647984, and NCT03945526).

The microalgae *Spirulina maxima*, administered as a food supplement, was proven effective against systemic arterial hypertension in a randomised pilot clinical trial, reducing blood pressure and decreasing markers of endothelial damage [94].

### 2.3. Marine Compounds Approved for CVDs Prevention

Omega-3-acid ethyl esters, derived from fish, are the only marine product marketed for CVD prevention so far. Approved by both the FDA and the EMA (in 2004 and 2005, respectively) for the reduction of triglyceride plasmatic levels in patients with severe hypertriglyceridemia, they are now commercialised by several brands [4,95].

## 3. Marine Compounds and Type 2 Diabetes Mellitus

Type 2 diabetes mellitus (T2DM) is a chronic multifactorial disease characterised by hyperglycaemia, due to defective insulin secretion and the occurrence of insulin resistance in the liver, skeletal muscle, and adipose tissue, often causing severe complications such as nephropathy, retinopathy, CVD, and disability [96,97]. During ageing, senescent cells accumulate in multiple organs, including pancreatic islets, which play a primary role in the regulation of blood glucose and lipid levels. Senescent β-cells display altered insulin production, leading to impaired glucose and lipid homeostasis, and strongly contributing to the pathogenesis of T2DM [98]. Hence, the incidence of T2DM rapidly increases with age. It was estimated that in 2019, 136 million elderly people (>65) live with diabetes worldwide (1 in 5), and that this number will reach 195.2 million by 2030 [99]. In addition to ageing, obesity is an important risk factor for T2DM since the over-secretion of pro-inflammatory adipocytokines by fat-enriched adipocytes is associated with the development of insulin resistance [100]. Thus, the treatment of diabetes is closely related to the alleviation of hyperlipidemic disorders, as for CVD.

Current anti-diabetic drugs act through different mechanisms. Inhibitors of α-glucosidase and α-amylase enzymes (e.g., acarbose) reduce the metabolism of polysaccharides into glucose/fructose in the intestine, thus controlling postprandial hyperglycaemia. The adenosine monophosphate-activated protein kinase (AMPK) and Akt pathways are targeted by other antidiabetics (e.g., metformin and berberine) to reduce hepatic gluconeogenesis and to induce insulin sensitivity and glucose uptake by skeletal muscle cells [101]. The peroxisome proliferator-activated receptor gamma (PPAR-γ) is activated by drugs such as rosiglitazone, resulting in increased insulin sensitivity and glucose uptake in adipose tissue and muscle and stimulating fatty acid oxidation. Sodium-Glucose Transport Protein 2 (SGLT2) inhibitors promote glucose urinary excretion. Inhibitors of the protein tyrosine phosphatase 1B (PTP1B), a negative regulator of the insulin signalling pathway, have emerged as a promising target for diabetes, but none have yet reached the market [102].

The chronic use of anti-diabetics may enhance their side effects, especially in elderly people, and this aspect may be further exacerbated by the presence of co-morbidities and polypharmacy, i.e., multi-drug consumption. Marine compounds may offer valuable alternatives. Indeed, several molecules with mechanisms related to glucose homeostasis were identified in vitro and have been extensively reviewed elsewhere [103,104,105,106].

### 3.1. Pre-Clinical Studies on Marine Compounds for T2DM Treatment

Several marine molecules tested in animal models of diabetes displayed anti-diabetic properties, primarily assessed as the ability to reduce blood glucose levels (hypoglycemic effects) (Table 2).

Dietary intake of different species of macroalgae (e.g., *Petalonia binghamiae*, *Padina arborescens*, *Ecklonia stolonifera*, *Ecklonia cava*, *Sargassum yezoense*, *Sargassum polycistum*, *Sargassum coreanum*, *Ulva rigida*), the seagrass Posidonia oceanica, and the soft coral Sinularia erecta, administered as extract or powder, displayed anti-diabetic properties in animal models, often associated with a hypolipidemic effect [107,108,109,110,111,112,113,114,115,116,117,118,119] (Table 2). In brown algae, such effects were suggested to be mediated by the highly abundant phlorotannins, polyphenolic compounds that demonstrated α-glucosidase and α-amylase inhibitory activity in vitro [117,120]. Dieckol, isolated from the brown algae Ecklonia cava, is a phlorotannin with a broad spectrum of biological properties, from anti-bacterial and anti-viral to anti-inflammatory and hypolipidemic [121]. This compound showed anti-diabetic effects in rat, mouse, and zebrafish models of diabetes, including a decrease in blood glucose, glycosilated haemoglobin and lipid levels, preservation of pancreatic β-cells, increased insulin production, and glucose tolerance. Its mechanisms of action involve α-glucosidase and α-amylase inhibition, as well as activation of the AMPK and Akt pathways [118,119,122,123,124]. Also, diphlorethohydroxycarmalol, a phlorotannin compound derived from the brown algae Ishige okamurae, exerted its hypoglicemic effect, observed in diabetic mice, through potent α-glucosidase and α-amylase inhibitory activity, stronger than acarbose [125]. Notably, acarbose, either alone or in combination with rapamycin, is among the three agents that have shown significant lifespan extension in both male and female mice, according to the Intervention Testing Programme (ITP) [126]. Therefore, diphlorethohydroxycarmalol holds particular interest in the context of its potential anti-ageing implications.

**Table 2:** Marine compounds showing antidiabetic effects in pre-clinical in vivo studies and related mechanisms of action.

Marine Compound	Source	Diabetic Model	Effects	Mechanisms	Positive Control	Ref.
Extract	Brown algae *Petalonia binghamiae*	Mice, stz-induced	Hypoglicemic, glucose tolerance increase	Pparg, glut4 and irs upregulation (adipocytes)	Rosiglitazone	[107]
Extract	Brown algae *Padina arborescens*	Mice, stz-induced	Hypoglicemic	A-glucosidase and α-amylase inhibition	Acarbose	[108]
Methanolic extract	Brown algae *Sargassum coreanum*	Db/db mice	Hypoglicemic, insulin response increase, hypolipidemic	Regulation of hepatic glycogen metabolism (↑ gck, ↓ g6pase, ↓ pepck, ↑ glicogen)	Rosiglitazone	[112]
Methanolic extract	Brown algae *Sargassum yezoense*	Db/db mice	Hypoglicemic, hypolipidemic	↓ g6pase (liver), Pparg, ucp3 and adiponectin upregulation (white fat)	Rosiglitazone	[113]
Water/ethanolic extracts	Brown algae *Sargassum polycistum*	Rats, stz-induced	Hypoglicemic, insulin response increase, hypolipidemic, liver, kidney and pancrease damage decrease	n.a.	Metformin	[114,115]
Extract	Green algae *Ulva rigida*	Rats, stz-induced	Hypoglicemic, hypolipidemic	Antioxidant		[116]
Methanolic extract (polyphenols-rich)	Brown algae *Ecklonia stolonifera*	kk-a(y) mice	Hypoglicemic	A-glucosidase inhibition, radical scavenging	Absent	[117]
Powder	Brown algae *Ecklonia cava*	Mice, stz-induced	Hypoglicemic, insulinotrophic (β-cell preservation, insulin secretion increase), hypolipidemic, liver steatosis improvement		Absent	[118]
Methanolic extract	Brown algae *Ecklonia cava*	Rats, stz-induced	Hypoglicemic, insulin production increase	Ampk and akt signalling activation	Absent	[119]
Dieckol-rich extract	Brown algae *Ecklonia cava*	db/db mice	Hypoglicemic, glucose tolerance increase, hypolipidemic	Regulation of hepatic glycogen metabolism (↑ gck, ↓ g6pase, ↓ pepck), antioxidant	Rosiglitazone	[109]
Methanolic extract	Soft coral *Sinularia erecta*	Rats, stz-induced	Hypoglicemic		Metformin	[110]
Extract	Seagrass *Posidonia oceanica*	Rats, alloxan-induced	Hypoglicemic, vasoprotective		Absent	[111]
Dieckol	Brown algae *Ecklonia cava*	Mice, stz-induced; zebrafish, alloxan-induced; db/db mice	Hypoglicemic	A-glucosidase and α-amylase inhibition, regulation of hepatic glycogen metabolism (↓ g6pase, ↓ pepck), ampk and akt signalling activation, antioxidant	Acarbose, metformin	[122,123,124]
Diphlorethohydroxycarmalol (DPHC)	Brown algae *Ishige okamurae*	Mice, Stz-induced	Hypoglicemic	A-glucosidase and α-amylase inhibition	Acarbose	[125]
Fucoxanthin	Brown algae *Undaria pinnatifida* *Laminaria japonica*	Mice, high fat diet-induced obese; kk-a(y) mice; db/db mice	Hypoglicemic, hypolipidemic, hypoglicemic, hyperinsulinemia suppression, hypolipidemic, insulin resistance improvement, pancreas damage decrease	Akt and ampk signalling activation, IR signalling activation, glut4 increase, adipocytokine reduction, regulation of glycogen metabolism (↑ gck, ↓ pepck, ↓ gsk3β, ↑ gsy)	Metformin	[127,128,129,130]
Fucoidan	Brown algae spp.	Db/db mice; gk rats; mice, stz-induced	Hypoglicemic, serum insulin decrease, pancreas damage decrease, glycosilated hb decrease	Camp pathway activation, sirt-1 activation, ampk/gapdh/pdx-1 signaling activation, α-glucosidase and α-amylase inhibition, NF-κb signaling inhibition, microbiota changes	Metformin, acarbose	[131,132,133,134,135,136,137,138,139]
Polysaccharides	Brown algae *Laminaria japonica*	Mice, alloxan-induced	Hypoglicemic, increased insulin levels, hypolipidemic		Glibenclamide	[140,141]
	Brown algae *Undaria pinnatifida*	Rats	Hypoglicemic, glucose tolerance increase, insulin sensitivity increase, liver and kidney damage decrease	Microbiota changes, AKT signalling activation, regulation of glycogen metabolism (↓ G6Pase, ↓ PEPCK)		[142]
	Red algae *Gracilaria lemaneiformis*	Mice, alloxan-induced	Hypoglicemic, kidney damage repair	Antioxidant	Acarbose	[143]
	Green algae *Enteromorpha prolifera*	Rats, stz-induced	Hypoglicemic, insulin sensitivity increase, pancreatic β-cells increase	↑GCK ↑ IR (liver), ↑ GLUT4 and adiponectin (adipose tissue)	Metformin	[144]
Sulfated polysaccharides	Brown algae *Undaria pinnatifida*	Mice, stz-induced	Hypoglicemic, glucose tolerance increase, insulin sensitivity increase, pancreatic islet preservation, liver steatosis decrease		Acarbose	[145]
Sulphated galactofucan	Brown algae *Undaria pinnatifida*	Mice, stz-induced	Slight hypoglicemic, slight hypolipidemic	Microbiota changes	Absent	[146]
Butyl-isobutyl-phthalate	Brown algae *Laminaria japonica*	Rats, stz-induced	Hypoglicemic	α-glucosidase inhibition		[147]
Octaphlorethol A	Brown algae *Ishige foliacea*	db/db mice	Hypoglicemic, improve glucose tolerance	AMPK and Akt signalling activation, ↑ GLUT4, regulation of glycogen metabolism (↓ G6Pase, ↓ PEPCK)	Absent	[148]
Bromophenol derivatives	Red algae *Rhodomela confervoides*	Rats, stz-induced	Hypoglicemic	Ptp1b inhibition	Absent	[149]
HPN (synthetic bromophenol derivative)	Red algae *Rhodomela confervoides*	db/db mice	Hypoglicemic, hypolipidemic	PTP1B inhibition	Rosiglitazone	[150]
Fucosterol	Brown algae *Pelvetia siliquosa*	Rats, stz-induced	Hypoglicemic	Aldose reductase and PTP1B inhibition	Metformin	[151,152]
Oligopeptides	Salmon skin *Oncorhynchus kern*	Rats, stz-induced	Hypoglicemic, Β-cell apoptosis decrease	Anti-inflammatory Antioxidant	Absent	[153]
Protein hydrolysate	Octopus muscle *Octopus vulgaris*	Rat, alloxan-induced	Hypoglicemic, insulin production increase, pancreas and liver damage decrease, hypolipidemic	A-amylase inhibition	Acarbose	[154]
Collagen peptides	Fish bone *Harpadon nehereus*	Mice, stz-induced	Hypoglicemic, insulin secretion increase, pancreas and liver damage decrease	Regulation of hepatic glycogen metabolism (↑ gck, ↑ gsk3β,↓ pepck, ↓ g6pase, ↑ glycogen)	Metformin	[155]
S-8300	Shark liver *Squalus mitsukurii*	Mice, stz-induced	Hypoglicemic, pancreatic islet damage decrease, hypolipidemic	Antioxidant	Glibenclamide	[156]
APSL (active peptide from shark liver)	Shark liver *Chiloscyllium plagiosum*	Mice, stz-induced	Hypoglicemic, insulin secretion and sensitivity increase, pancreatic islet preservation, hypolipidemic, liver steatosis decrease, pancreas, liver and kidney damage decrease	Anti-inflammatory	Metformin	[157]

STZ—streptozotocin; GCK—glucokinase; G6Pase—glucose 6-phosphatase; PEPCK—phosphoenolpyruvate carboxykinase; PPARg—peroxisome proliferator activated receptor gamma; UCP3—uncoupling protein 3; GLUT4; GSK3β—glycogen synthase kinase-3 beta; GYS—glycogen synthase; AMPK—AMP-activated protein kinase; PTP1B—protein tyrosine phosphatase 1B; GLUT4—glucose transporter 4; IR—insulin receptor; PDX-1—pancreatic and duodenal homeobox 1.

Dietary fucoxanthin, a specific carotenoid present in the chloroplasts of brown algae, exerts a significant anti-diabetic and anti-obesity effect in models of diabetic/obese mice, regulating blood glucose, improving insulin resistance, and reducing body and adipose tissue weight [158]. The main mechanisms mediating such effects suggested by the different in vivo studies include upregulation of insulin receptor and Akt signalling in liver and skeletal muscle [128,130], induction of the glucose transporter type 4 (GLUT-4) in skeletal muscle [127,128,130], reduced secretion of adipokines involved in insulin resistance [129], regulation of glycogen synthesis [130], and decreased white adipose tissue (while increasing brown one) [129]. The hypolipidemic effect of fucoxanthin may be mediated by the induction of the uncoupling protein 1 (UCP1) (a mitochondrial protein typical of brown fat) in white fat, thus stimulating fatty acid oxidation, dissipating energy through heat production, and reduce lipid excess in adipocites [158,159].

Other algal compounds displayed hypoglicemic effects in vivo: butyl-isobutyl-phthalate, a potential α-glucosidase inhibitor [147,160], octaphlorethol A, acting through activation of the AMPK and Akt pathways [148], bromophenol-derived compounds from red alga *Rhodomela confervoides* [149], and fucosterol [151], which both demonstrated PTP1B inhibitory activity [149,150,152,161].

Macroalgae are also particularly rich in polysaccharides exclusive to the marine environment. Among them, fucoidan has a widely studied role in the treatment of diabetes and its complications, e.g., diabetic nephropathy and retinopathy [24]. A substantial number of studies have demonstrated that treatment with fucoidan (extracted from various algal sources) is effective in lowering hyperglycaemia, regulating glucose metabolism, increasing insulin production, and alleviating pancreatic β-cell damage [24]. Fucoidan showed α-amylase and α-glucosidase inhibitory effects in vitro, and various other mechanisms of action were suggested, e.g., the activation of cAMP and Akt signalling pathways [24]. To note, it was demonstrated in vivo that the molecular mechanisms underlying the increase in insulin synthesis by fucoidan may include activation of Sirt-1-dependent upregulation of PDX and GLP-1R [134]. Moreover, fucoidan modulates the gut microbiota of diabetic mice, decreasing the abundance of intestinal bacteria associated with the development of diabetes [138,139]. In addition, the aforementioned hypolipidemic and anti-hypertensive effects of fucoidan were observed even in diabetic mice [162]. Recently, it was demonstrated in a T1DM mouse model that fucoidan supplementation reduces blood glucose levels and modifies the pancreatic microenvironment, enhancing autophagy and apoptosis of pancreatic β cells by the AMPK/mTOR1/TFEB signalling pathway [163].

Other algal polysaccharides were effective in lowering blood glucose. Sulfated polysaccharides derived from the brown seaweed *Undaria pinnatifida* (e.g., sulfated galactofucan) mitigated insulin resistance, improved glucose tolerance and dyslipidemia, and reduced hepatic steatosis and damage to pancreatic islets, liver, and kidney. Such improvements were associated with changes to the intestinal microbiota [142,145,146]. In diabetic mice, the hypoglycemic effect of polysaccharides from *Laminaria japonica* was observed together with increased insulin and lipid levels in serum [140,141], while an unidentified polysaccharide from the red algae *Gracilaria lemaneiformis*, alleviating hyperglycaemia and pancreas and kidney tissue damage, likely acts thanks to its antioxidant effects [143]. *Enteromorpha prolifera* polysaccharides significantly increased the number of pancreatic β-cells and enhanced insulin sensitivity in diabetic rats; such effects were mediated by the increase of hepatic glucokinase (GCK) and insulin receptor (IR), enhanced GLUT-4 and adiponectin (APN) in the adipose tissue, and antioxidant action [144].

Functional peptides were also studied for their potential use in diabetes treatment. Oligopeptides from marine salmon skin [164], protein hydrolysates from octopus muscle [154], and collagen peptides from fish bone [155] were shown to drive an anti-diabetic effect upon oral administration, associated with an improvement of pancreas and/or liver damage. Anti-inflammatory and antioxidant properties [164], α-amylase inhibition [154], and regulation of hepatic glycogen metabolism (decreased glucose 6-phosphatase, G6Pase, and phosphoenolpyruvate carboxykinase, PEPCK1; increased GCK and phosporylation of glycogen synthase kinase-3β, GSK3β) [155] were detected as possible target mechanisms. The Active Peptide from Shark Liver (APSL) and S-8300, hepatic functional peptides isolated from shark species, were found to lower hyperglycaemia in diabetic mice, increasing insulin secretion and sensitivity, repairing lesions to pancreatic islets, and having a hypolipidemic effect [156,157]. In addition, APSL improved hepatic steatosis and damage to liver and kidney to a greater extent than metformin and highlighted its anti-inflammatory properties [157]. Further studies suggested that the APSL is located at the N-terminus of the TBC1D15 protein [165], which is involved in GLUT4 vesicular trafficking and membrane translocation [166], thus providing an additional mechanism of action.

### 3.2. Marine Compounds under Clinical Trial for T2DM Treatment

Despite the numerous and promising pre-clinical studies on marine compounds with anti-diabetic properties, the clinical trials are still quite rare, and none of these molecules have reached the market so far. Moreover, clinical investigations have not always matched the promising effects seen in preclinical experiments.

Oral administration of fucoidan to obese non-diabetic patients did not affect glycemia, insulin levels, or insulin resistance compared to controls (ACTRN12614000495628) [167], and no effect of a fucoidan-rich extract from the algae *Ascophyllum nodosum* was observed on postprandial glucose levels or insulin response in normoglycemic subjects (NCT05460884) [168]. Another clinical study with fucoidan treatment is ongoing on pre-diabetic individuals (ACTRN12621000413820) [169], which will allow us to define its antidiabetic potential in a more clinically relevant population. A study including healthy participants on the impact of polyphenol-rich brown seaweed (*Fucus vesiculosus*) extract showed no effect on glycemia or insulin levels (ACTRN12616000126415p) [170], as well as the intake of the *Undaria pinnatifida* algae (containing fucoidan, fucoxanthin, and polyphenols) in a healthy population, while a hypoglycaemic effect was observed after administration of its sporophylls, likely due to the higher fibre content [171]. A slight effect was also observed after administration of hydrolysates of the tunicate *S. clava* in patients with Type 2 diabetes and hypertension, where significant reductions in blood pressure and glycosilated haemoglobin were observed, with a non-significant decrease in insulin and insulin resistance [172].

Conversely, an effective anti-diabetic activity was observed for a dieckol-rich extract from the brown algae *Ecklonia cava*, which reduced hyperglycaemia and insulin resistance in a randomised clinical trial enrolling pre-diabetic subjects without evident side effects after dietary supplementation [173]. Similarly, marine collagen peptides significantly reduced levels of fasting blood glucose, insulin, glycosilated haemoglobin, and lipids while increasing insulin sensitivity in Type 2 diabetic patients [153]. The efficacy of the microalgae *Chlorella vulgaris* in the treatment of hyperglycaemia and dyslipidaemia was demonstrated in a randomised trial including patients with non-alcoholic fatty liver disease (which often coexists with diabetes [174]), which displayed a significant decrease in fasting serum glucose levels and in body weight (201202233320N7) [175]. Fucoxanthin effectively increases insulin secretion and decreases body weight, blood pressure, and triglycerides in patients with metabolic syndrome (NCT03613740) [176].

Clinical results on the effect of fish omega-3 on glucose-insulin homeostasis are controversial. A meta-analysis including 20 randomised clinical trials on the effects of omega-3 in Type 2 diabetic patients revealed no significant changes for plasmatic glucose, glycosilated haemoglobin, body weight, or serum lipids, except for triglycerides, which were significantly decreased [177]. Contrasting results may be attributed to a sex-specific response bias [178]. No effect of omega-3 was reported in T2DM patients for the prevention and treatment of chronic kidney disease, a common T2DM complication (NCT01684722) [179].

## 4. Discussion

**Figure 1:**
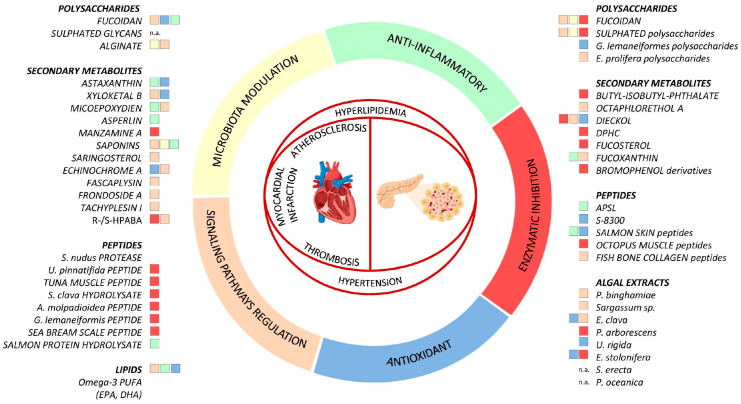
Main mechanisms of action of marine compounds targeting CVD and T2DM in vivo.

Marine compounds have a strong potential for the management of age-related diseases. This review highlighted the wide burden of promising compounds for the treatment and prevention of CVD and diabetes. Due to the close link between such pathologies and the high risk of comorbidity, the development of drugs with pleiotropic effects is of outmost interest. Compounds with an action on common factors, like hyperlipidaemia, inflammation, and oxidative stress, could probably have a therapeutic role in both diseases. Several mechanisms have been suggested for marine compounds targeting CVDs and T2DM (Figure 1).

Molecules that specifically target a single enzyme (e.g., ACE, PTPB1, α-amylase, and α-glucosidase inhibitors) or signalling pathway (e.g., FGF2, P2Y_12_, and insulin pathway) are expected to be effective for a restricted pathological condition. Molecules with antioxidant effect (e.g., fucoidan, xyloketal B, echinochrome A, dieckol, and S-8300 peptide) and anti-inflammatory action (e.g., fucoidan, astaxanthin, fucoxanthin, micoepoxydien, asperlin, saponins, and APLS peptide) may potentially hold a wider therapeutic spectrum. Anti-inflammatory properties are particularly relevant since ageing is associated with low-grade chronic inflammation, which is one of the underlying causes of many age-related diseases [180,181].

Molecules that induce changes in the microbiota composition, mainly algal polysaccharides (e.g., fucoidan, potassium alginate, sulphated galactofucan, and polysaccharides from *Undaria pinnatifida*) and sea cucumber saponins, represent interesting candidates for drugs with pleiotropic effects. Indeed, the gut microbiota is involved in the regulation of multiple physiological and pathological processes; its age-related changes contribute to health loss in the elderly and to several non-communicable pathologies [182], and its pharmacological modulation may potentially impact global individual health.

Different marine drugs have been shown to target mitochondria or mitochondrial signalling pathways, which may help to counteract pathological processes not only in metabolic and cardiovascular diseases but also in neuro-muscular age-related diseases [40].

Fucoidan was shown to be the molecule that displayed more beneficial effects, being able to target different diseases, from atherosclerosis, thrombosis, and hypertension (among CVD) to diabetes. This may be partly due to its broad effects on inflammation, oxidative stress, and gut microbiota (Figure 1) and partly biased by the fact that fucoidan is one of the most common and studied marine compounds compared to the others. Further studies would be useful to elucidate the possible role of other marine compounds in the co-treatment of CVD and T2DM.

However, we also highlighted evident limits in translating the knowledge from preclinical research to clinical use. Indeed, to date, only one marine molecule among the many candidates has reached the market, namely the omega-3 acid ethyl esters for the treatment of hypertriglyceridemia as a preventative measure for CVD. The availability of the compound of interest is a challenging issue since extraction from the natural source often provides a low yield, which is a limit for industrial and pharmaceutical applications. In addition, while algae and marine microorganisms may be cultivated, the exploitation of marine animals is not desirable due to important ecological implications. Further, the isolation of compounds from the natural environment may include the risk of contamination by chemical pollutants (e.g., heavy metals and hydrocarbons) and other emerging contaminants (e.g., microplastics, endocrine disruptors, and pharmaceuticals), which are widespread in almost all marine ecosystems. The development of synthetic strategies for high-yield production is essential to overcome these limitations. Methods of chemical synthesis may be applied for the production of analogues of natural compounds with the same or enhanced biological activity and pharmacokinetic properties [150,183]. The availability of functional peptides would be significantly improved by the production of recombinant versions, as developed for the active peptides from shark liver (APSL) [184]. The production of synthetic or recombinant analogues may enhance the availability of marine-derived molecules and also overcome the difficulty of patenting natural substances, thus increasing the interest of pharmaceutical companies. Further, various marine-derived compounds, such as fucoidan, sulfated polysaccharides, and functional peptides, are still lacking standardisation and quality control measures to ensure the constant purity and bioactivity of these compounds, which are crucial considerations for their therapeutic use. In many cases, the lack of chemical characterization of the active compound in an algal extract or fraction hinders the transition from animal to human studies. The absence of a positive control in pre-clinical evaluation (Table 1 and Table 2) sometimes limits the assessment of the real benefit of the marine compound alternative compared to the gold standard therapy. Moreover, longitudinal studies in aged mice with an appropriate sample size, a blinded design, and strong functional [185] as well as molecular [186] health outcomes are still lacking. In the end, although various potential mechanisms of action for marine-derived compounds have been proposed, there is a need for more in-depth investigation of the underlying molecular mechanisms and pathways involved. Providing more detailed insights into how these compounds exert their effects at the cellular and molecular levels would enhance their translation into clinical trials.

## 5. Material and Methods

PubMed, Scopus, and Epistemic AI databases were used for the bibliographic search. For the first search, the keywords “marine compounds” or “marine molecules” were combined with words related to the pathologies of interest (e.g., “cardiovascular disease”, “atherosclerosis”, “thrombosis”, “myocardial infarction”, “hypertension”, “diabetes”, and “hyperglycemia”). In a second step, the keywords related to specific marine compounds identified with the first search (e.g., “fucoidan”, “alginate”, “astaxanthin”, “dieckol”, “marine peptides”, etc.) were combined to the terms “pre-clinical studies”, “in vivo studies”, “clinical studies”, or “human studies”. The search was updated until February 2024.

## 6. Conclusions

Despite the wide and promising pool of marine-derived molecules displaying pharmacological properties against CVD and T2DM, the path towards their clinical application is still long. Many of them are of particular interest in the context of ageing, since they act on targets highly relevant to the ageing process, such as inflammation, oxidative stress, and gut microbiota. Their minimal side effects and the potential to act with a pleiotropic effect are also optimal characteristics for use in the elderly, which often require a chronic assumption of multiple drugs.
